# Administration of *Bifidobacterium breve* PS12929 and *Lactobacillus salivarius* PS12934, Two Strains Isolated from Human Milk, to Very Low and Extremely Low Birth Weight Preterm Infants: A Pilot Study

**DOI:** 10.1155/2015/538171

**Published:** 2015-02-22

**Authors:** Laura Moles, Esperanza Escribano, Javier de Andrés, María Teresa Montes, Juan M. Rodríguez, Esther Jiménez, Miguel Sáenz de Pipaón, Irene Espinosa-Martos

**Affiliations:** ^1^Departamento Nutrición, Bromatología y Tecnología de los Alimentos, Universidad Complutense de Madrid, 28040 Madrid, Spain; ^2^Servicio de Neonatología, Hospital Universitario La Paz, 28046 Madrid, Spain; ^3^Probisearch, Tres Cantos, 28760 Madrid, Spain

## Abstract

The preterm infant gut has been described as immature and colonized by an aberrant microbiota. Therefore, the use of probiotics is an attractive practice in hospitals to try to reduce morbidity and mortality in this population. The objective of this pilot study was to elucidate if administration of two probiotic strains isolated from human milk to preterm infants led to their presence in feces. In addition, the evolution of a wide spectrum of immunological compounds, including the inflammatory biomarker calprotectin, in both blood and fecal samples was also assessed. For this purpose, five preterm infants received two daily doses (~10^9^ CFU) of a 1 : 1 mixture of *Bifidobacterium breve* PS12929 and *Lactobacillus salivarius* PS12934. Bacterial growth was detected by culture-dependent techniques in all the fecal samples. The phylum *Firmicutes* dominated in nearly all fecal samples while *L. salivarius* PS12934 was detected in all the infants at numerous sample collection points and *B. breve* PS12929 appeared in five fecal samples. Finally, a noticeable decrease in the fecal calprotectin levels was observed along time.

## 1. Introduction

The gut microbiota of preterm infants is usually described as aberrant when compared to that of healthy term infants. Very often, the former is characterized by a notably lower bacterial diversity, a lower presence of bifidobacteria, and a higher concentration of potentially pathogenic bacteria [1–7]. This may have short-, medium-, and long-term health consequences since early colonizing organisms interact with the intestinal mucosa to shape the developing immune system [8, 9].

In fact, interactions with different components of the microbiota are crucial to the establishment and development of T-cell subsets, including NK, Treg, and Th17 cells, in the appropriate proportions to achieve homeostasis [10].

Many preterm infants lack an important part of transplacental transfer of maternal antibodies since this process occurs mainly in the last third of pregnancy; in addition, they have an impaired pattern-recognition receptor function and a reduced leukocyte endothelial adhesion and extracellular bacterial elimination [11]. Together, these alterations in the microbial colonization pattern and in the maturation of immune system, together with their stay in a hospital environment and other factors, predispose preterm infants to infections and/or to diseases such as necrotizing enterocolitis (NEC) [12–15].

The administration of probiotics to preterm neonates often leads to a decrease in the morbidity and mortality rates, in those of NEC and, in some cases, even in those of sepsis [16–22]. Additional benefits associated with probiotic supplementation in preterm neonates include earlier achievement of full enteral feeding [22], a lower colonization by* Enterobacteriaceae* [23], and a better neurological and immunological evolution [22, 24]. For these reasons, the number of institutions including probiotic supplementation in routine preterm care is increasing rapidly although the safety of probiotics in very low and extremely low birth weight infants is still a matter of debate [25], the mechanisms backing such effects are not well known yet [10], and global conclusions are difficult to establish because different studies usually make use of different probiotic strains, dosages, and/or treatment period.

Human milk is acknowledged as the best feeding option to preterm infants [26, 27] because its use decreases the incidence of many negative outcomes of prematurity, such as late onset sepsis or NEC [28–30]. In addition, human milk seems to be an important source of potentially beneficial bacteria to the infant gut and some strains may find future applications as probiotics for preterm infants [31–36]. In this context, the objective of this exploratory study was to assess early gut colonization in a short cohort of preterm neonates receiving a combination of two probiotic strains isolated from human milk. Furthermore, a wide variety of blood and fecal immunological parameters were assessed in order to elucidate their utility in future studies involving a larger cohort.

## 2. Materials and Methods

### 2.1. Study Design and Sampling

Five preterm infants were enrolled in this study within 2 days after their birth. All of them met the following inclusion criteria: birth weight < 1,300 g, gestational age at birth < 29 weeks, and absence of any malformation or metabolic disease at birth. The most relevant demographic and clinical variables from mother-infant pairs were compiled by the Medical Staff of the Service of Neonatology of the Hospital Universitario La Paz (Madrid, Spain). The Ethical Committee on Clinical Research of the Hospital Universitario La Paz of Madrid approved all study protocols (code number: 3551). Samples and clinical information were obtained after written informed consent by the infants' parents. This trial is registered with ClinicalTrials.gov identifier NCT02192996.

After spontaneous meconium expulsion (between the second and the fourth days of life), a mixture of* Bifidobacterium breve* PS12929 and* Lactobacillus salivarius* PS12934, containing ~1 × 10^9^ colony-forming units (CFU) of each strain, was suspended in a sterile saline solution and administered twice a day to the infants through an enteral feeding system. Meconium samples were collected prior to probiotic administration and, later, fecal (*n* = 14) and blood (*n* = 10) samples were collected weekly for up to 28 days. Fecal samples were aliquoted and stored at −80°C or −20°C until microbiological or immunological analysis, respectively. Blood samples were collected in ethylenediaminetetraacetic acid (EDTA) tubes; subsequently, the plasma was obtained within 4 h after extraction and stored at −20°C until analysis.

### 2.2. Microbiological Analysis

Adequate dilutions of five meconium and fourteen stool samples were spread onto Kanamycin Aesculin Azide Agar (KAA; Oxoid) for* Enterococcus* species isolation; de Man, Rogosa and Sharpe (MRS; Oxoid, Basingstoke, UK) supplemented with L-cysteine (0.5 g/L) (Sigma, St. Louis, USA) (MRScys) for isolation of lactic acid bacteria; MacConkey (MCK; BioMérieux, Marcy l'Etoile, France) for isolation of* Enterobacteriaceae;* Sabouraud Dextrose Chloramphenicol (SDC, BioMérieux) for isolation of yeasts; TOS-Propionate (TOS; Merck, NJ, USA) for isolation of bifidobacteria; and Columbia Nalidixic Acid Agar (CNA, BioMérieux) as a general medium for isolation of other bacterial groups. Plates were aerobically incubated at 37°C for up to 48 h, with the exception of MRScys and TOS plates that were anaerobically incubated (85% nitrogen, 10% hydrogen, and 5% carbon dioxide) in an anaerobic workstation (Mini-MACS Don Whitley Scientific Limited, Shipley, UK) at 37°C for 48 h. Bacterial counts were recorded as the CFU/g of meconium or feces and transformed to log_10_ values before statistical analysis.

At least one representative of each different colony type obtained from each sample was isolated. Approximately 140 isolates were analyzed by optical microscopy and identified by MALDI-TOF mass spectrometry in a Vitek-MS instrument (BioMérieux, Marcy l'Etoile, France) in the facilities of Probisearch S. L. (Tres Cantos, Spain).

Pulsed-field gel electrophoresis (PFGE) genotyping of all the isolates identified as* L. salivarius* or* B. breve* was carried following a protocol previously described [37]. The profiles were compared to those of* L. salivarius* PS12934 and* B. breve* PS12929, respectively.

### 2.3. Immunological Analysis

The concentration of 18 cytokines, chemokines, and growth factors, including interleukin (IL) IL-1_*β*_, IL-6, IL-12 (p70), interferon-*γ* (INF-*γ*), tumor necrosis factor-*α* (TNF-*α*), IL-2, IL-4, IL-10, IL-13, IL-17, IL-8, growth related oncogene-*α* (GRO-*α*), macrophage-monocyte chemoattractant protein-1 (MCP-1), macrophage inflammatory protein-1_*β*_ (MIP-1_*β*_), IL-5, IL-7, granulocyte colony stimulating factor (G-CSF), and granulocyte-macrophage colony stimulating factor (GM-CSF), was determined in 5 meconium, 14 feces, and 10 plasma samples by using a Bio-Plex 200 system instrument (Bio-Rad, Hercules, CA) and the Bio-Plex Pro Human Cytokine, Chemokine and Growth Factor Assays (Bio-Rad). Parallel, the concentration of immunoglobulin (Ig) IgG_1_, IgG_2_, IgG_3_, IgG_4_, IgM, and IgA was determined using the Bio-Plex Pro Human Isotyping Assay Kit (Bio-Rad).

Before analysis, 0.1 g of meconium and fecal samples was diluted in 0.9 mL of peptone water, homogenized, and centrifuged for 15 min at 14,000 ×g at 4°C; then, supernatants (≥200 *μ*L) were collected. Plasma samples were defrosted and properly diluted immediately before the immunological assay. Analyses were carried out in duplicate following the manufacturer's protocol and standard curves were performed for each analyte. Lower limit of quantification (LLOQ) was different for each one of the parameters, ranging from 0.02 to 11.74 ng/L for cytokines and from 0.01 to 2 ng/L for immunoglobulins.

Additionally, calprotectin levels (LLOQ: 8 ng/L) were determined in 5 meconium, 14 feces, and 8 plasma samples using a commercially available enzyme-linked immunosorbent assay (ELISA) kit (Calpro, Lysaker, Norway) according to the manufacturer's instructions. The standard curve of calprotectin was obtained from triplicates of each assayed concentration and fit to a 4-parameter curve model.

### 2.4. Statistical Analysis

The statistical analysis was performed using R 2.15.3 (R-project, http://www.r-project.org). When data were not normally distributed, medians and interquartile ranges (Q1 and Q3) were calculated for all sampling times, and means and 95% confidence interval (95% CI) were used for normally distributed data. The richness and diversity of meconium and fecal microbiota were determined by calculating the Shannon-Weaver diversity index, which takes into account the number and evenness of the bacterial species. The Kruskal-Wallis test for nonnormal data or one-way ANOVA test, when data were normally distributed, was used to evaluate the differences between sampling times, in all measured variables, in plasma samples and for the comparison of immunological variables between plasma and fecal samples. The nonparametric Friedman test or one-way ANOVA test, when data were normally distributed, was used in fecal samples to evaluate the differences between sampling times in all measured variables. In all cases,* P* values of <0.05 were considered to be significant. Redundancy analysis (RDA) was used for exploration of whole data sets and evaluation of the possible relationship between gut colonization and immunological parameters with the clinical status of the participants. Finally, heatmaps of plasma and fecal samples were plotted. To do this, calculation of Kendall's correlation coefficients was performed and Ward agglomeration methods were used to obtain the clustering of the variables and cases matrix.

## 3. Results

### 3.1. Demographic and Clinical Characteristics of the Parti****cipants

The clinical and demographic data of the mothers and infants who participate in this study are summarized in Table 1. Although five preterm infants were included in this study, there were 2 sets of twins (infants 1 and 2; infants 3 and 4) and, therefore, data were collected from three mothers (Table 1).

All the infants were female and were born by Cesarean section with a mean gestational age of 28 weeks and 2 days. The mean birth weight was 1,020.4 g and the mean height and head circumference were 34.5 cm and 25.0 cm, respectively. These parameters showed* Z*-scores < 0. Infants stayed in the NICU a mean time of 30.6 days with a mean age at discharge of 65.4 days, which represented a mean corrected gestational age of 37 weeks and 5 days (Table 1). Additional information of clinical features is provided as supplemental information (Supplemental Information 1; see Table S1 of the Supplementary Material available online at http://dx.doi.org/10.1155/2015/538171).

### 3.2. Microbiological Analysis

Bacterial growth was detected in one meconium sample and in all the fecal samples. Differences in the bacterial counts of fecal samples were evaluated by nonparametric Friedman test on days 7, 14, 21, and 28 (data not shown).

**Figure 1:**
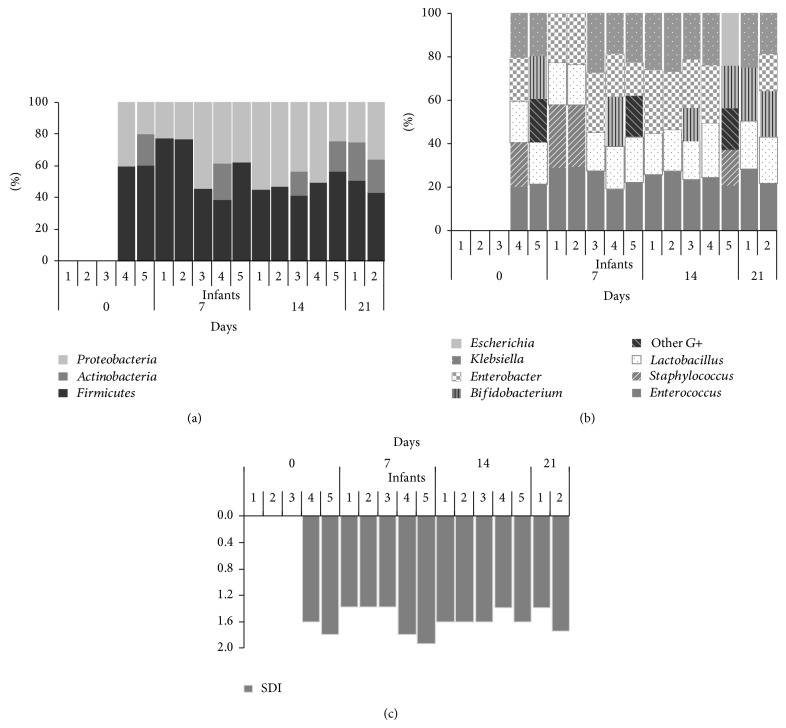
Phyla (a), genera (b), and bacterial diversity assessment by the SDI (c) of the microbiota of the meconium and fecal samples analyzed in this study. The relative contributions of the phyla and genera to the microbiota of the infant's gut and the SDI values were labeled per case and sampling time.

Globally, the phylum* Firmicutes* predominated in all the fecal samples except in those belonging to infant 5 where* Proteobacteria* was present in a similar proportion (Figure 1(a)). On the other hand,* Proteobacteria* dominated at the 14th day of intervention in fecal samples of the siblings 3 and 4. The phylum* Actinobacteria*, mainly represented by the genus* Bifidobacterium*, was isolated from day 7 although not in all the fecal samples (Figure 1(a)).

Among the* Firmicutes*, the genera* Enterococcus* and* Lactobacillus* were isolated from all the fecal samples except in that of infant 2 at day 21 where* Lactobacillus* could not be detected. The bacterial counts of* Enterococcus* decreased significantly from day 7 to day 21 of treatment (*P* = 0.043) from 10.00 to 8.30 log CFU/g. In contrast,* Lactobacillus* counts increased from 6.60 log CFU/g after 7 days of probiotic treatment to 8.32 log CFU/g at the end of the intervention; in this case, the differences were not statistically significant due to both the individual variability and the small cohort. The genus* Staphylococcus* was mainly isolated in the first weeks of the study from meconium and 7-day fecal samples (Figure 1(b)) with median counts of 4.30 and 9.44 log CFU/g, respectively.

In relation to* Proteobacteria*, the genus* Enterobacter* was isolated from all the fecal samples except from two from infant 2 (days 7 and 21) and from one of infant 3 at day 28 (Figure 1(b)). Similarly, the genus* Klebsiella* was isolated from all fecal samples except from two collected at day 7 (siblings 3 and 4) and one at day 21 (infant 2). Bacterial counts of these two genera were significantly different at every sampling day (*P* = 0.007 and 0.046 for* Enterobacter* and* Klebsiella*, resp.) and a decrease was observed in* Klebsiella* median counts (from 10.19 log CFU/g at day 7 to 8.48 log CFU/g at day 28).

Finally, the* Bifidobacterium* median counts oscillated between 7.98 and 9.98 log CFU/g in the 6 fecal samples where this genus was detected (Figure 1(b)).

The SDI of the fecal samples fluctuated during the study probably due to the different antibiotic treatments that the infants received (Figure 1(c)).

In order to detect the presence of* L. salivarius* PS12934 and* B. breve* PS12929 in fecal samples, all the fecal isolates belonging to such species were PFGE genotyped. This technique revealed that* L. salivarius* PS12934 was present in all the infants at numerous sampling points while* B. breve* PS12929 could be detected after day 14.

The heatmap obtained from the fecal samples at different sampling times of all the infants is shown in Figure S1. The dendrogram resulted after Kendall correlation coefficient calculation highlights the similar species profile of fecal samples of infant 2 at different sampling times and the almost identical species profile of fecal samples from days 7 and 14 of twins 3 and 4.

### 3.3. Immunological Analysis

A wide range of immune compounds were analyzed in plasma and fecal samples of the preterm infants throughout the study. An exploratory screening, using a principal component analysis (PCA) to detect outliers, revealed that the 7th day fecal sample from infant 4 was very different from the rest of the sample sets (data not shown). This infant was suffering a gastric bleeding at this sampling time and, therefore, this sample was excluded from the results of data sets.

Median values of the immune compounds concentrations in meconium and, also, in fecal samples at 7th and 14th days of probiotic supplementation are shown in Table 2. In general, the values obtained for all the immune factors showed a high interindividual variability in both detection frequencies and amounts. The levels of some immune compounds changed throughout the study; those of IgG_2_ and MCP-1 decreased progressively (*P* = 0.074 and *P* = 0.076, resp.) while that of IgA increased (>50 times) from meconium to fecal samples obtained at day 7 after birth (*P* = 0.074) (Table 2). However, only the inflammatory biomarker calprotectin decreased significantly along sampling time (*P* = 0.041).

Plasma concentrations of the immune compounds are shown in Table 3 and, as it can be observed, no significant changes were found. Globally, chemokines and proinflammatory compounds tended to decrease, with the exception of IL-12 and TNF-*α*. The levels of the latter and those of the anti-inflammatory compounds remained very constant along time. Plasma immunoglobulins also showed a high individual variability although all decreased, with the exception of IgG4 and IgM (Table 3).

The plasma concentrations of the different immune compounds were compared with their respective fecal values. All the immunoglobulins, with the exception of IgA, were significantly different in both types of samples. Among the remaining immune parameters, calprotectin, IL-10, GRO-*α*, and GM-CSF were significantly higher in feces (*P* = 0.000, *P* = 0.045, *P* = 0.048, and *P* = 0.000, resp.) while IL-8, MCP-1, and MIP-1_*β*_ were more abundant in plasma (*P* = 0.012, *P* = 0.000, and *P* = 0.001, resp.) (Table S2).

### 3.4. Multivariate Analysis of the Studied Population

A multivariate analysis was performed for investigating the possible relationship between clinical features and the immunological and microbiological profiles of fecal and plasma samples. The clinical variables considered were the following: antibiotherapy (Antibiotics); air way resume (AWResume) including ventilation, caffeine, and surfactant treatment; C-RP; hemoglobin amounts (Hb); hematocrit percentage (Hcte); ibuprofen treatment (Ibu.T); ibuprofen doses (Ibu.doses); number of stools per day (N°.stools); nutrition resuming the median feeding type (Nutrition); patent ductus arteriosus (PDA); Sepsis; spontaneous stools (Spont.stools); Transfusion; and Weight.

**Figure 2:**
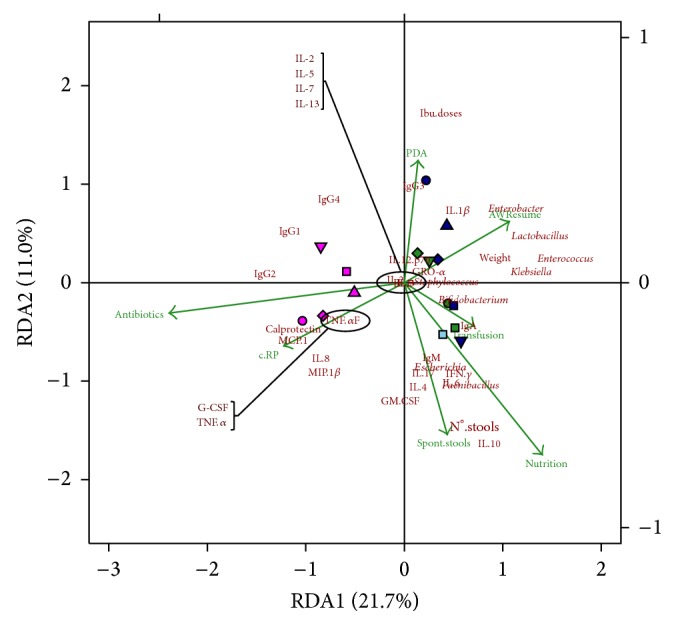
Redundancy analysis of the fecal samples obtained at different sampling times from the preterm infants. Cases were represented with points and then labeled per infant (1: circle, 2: square, 3: diamond, 4: triangle, and 5: inverted triangle) and sampling time (0: medium violet red, 7: green, 14: midnight blue, and 21: sky blue) Quantitative variables matrix, including the hematological and immunological parameters, ibuprofen doses (Ibu.doses), number of stools per day (N°.stools), and weight, was represented with each variable name or abbreviator in dark red color; clinical categorized observations vectors matrixes were used as constrained variables (airway resume (AWResume), antibiotherapy (Antibiotics), C-RP, ibuprofen treatment (Ibu treatment), nutrition type (Nutrition), patent ductus arteriosus (PDA), Sepsis, spontaneous stools (Spont.stools), and Transfusion) and represented as vectors in green color. The bidimensional RDA plot explains the 33% of the variability and showed a *P* value of 0.020 after 299 permutations when ANOVA test of the model was performed.

The redundancy analysis (RDA) of the above-mentioned variables for fecal samples is shown in Figure 2. The obtained model explains the 33% of the variability and the ANOVA test of the model was statistically significant (*P* = 0.020). The meconium samples were located opposite to microbial growth and in coincidence with the constrained antibiotic vector. Although the rest of fecal samples showed a less clear separation, the evolution of microbial colonization can be observed along the RDA1 axis in coincidence with the constrained vectors for AWResume, Nutrition, Spont.stools, PDA, and Transfusion and in opposite not only with the antibiotics and C-RP vectors, but also with the coordinates of proinflammatory compounds, such as calprotectin, MCP-1, MIP-1_*β*_, TNF-*α*, and IL-8 (Figure 2).

**Figure 3:**
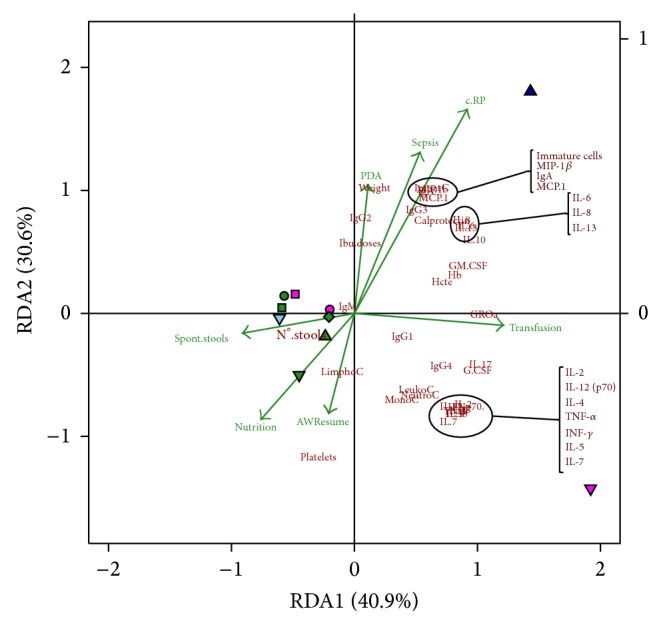
Redundancy analysis of the blood samples obtained at different sampling times from the preterm infants. Cases were represented with points and then labeled per infant (1: circle, 2: square, 3: diamond, 4: triangle, and 5: inverted triangle) and sampling time (0: medium violet red, 7: green, 14: midnight blue, and 21: sky blue) Quantitative variables matrix, including the hematological and immunological parameters, ibuprofen doses (Ibu.doses), number of stools per day (N°.stools), and weight, was represented with each variable name or abbreviator in dark red color; clinical categorized observations vectors matrixes were used as constrained variables (airway resume (AWResume), antibiotherapy (Antibiotics), C-RP, ibuprofen treatment (Ibu treatment), nutrition type (Nutrition), patent ductus arteriosus (PDA), Sepsis, spontaneous stools (Spont.stools), and Transfusion) and represented as vectors in green color. The bidimensional RDA plot explains the 71% of the variability and showed a *P* value of 0.010 after 199 permutations when ANOVA test of the model was performed.

The RDA of plasma samples (Figure 3) explains the 70% of the variability and the ANOVA test of the model was statistically significant (*P* = 0.010). The bidimensional plot shows two points clearly separated from the others: infant 4 at day 19 and infant 5 at day 7. Three different situations were observed; on the one hand coordinates from infants 1, 2, and 3 did not change among sampling times, while on the other infant 5 showed a normalization far away of proinflammatory variables and hematological parameters coordinates; and finally infant 4 that initially was close to her corresponding twin and the rest of participants appeared at day 19, in the positive RDA1 and RDA2 coordinates, related to constrained variables vectors corresponding to C-RP, Sepsis, and PDA reflecting the clinical worsening of this infant at this moment.

**Figure 4:**
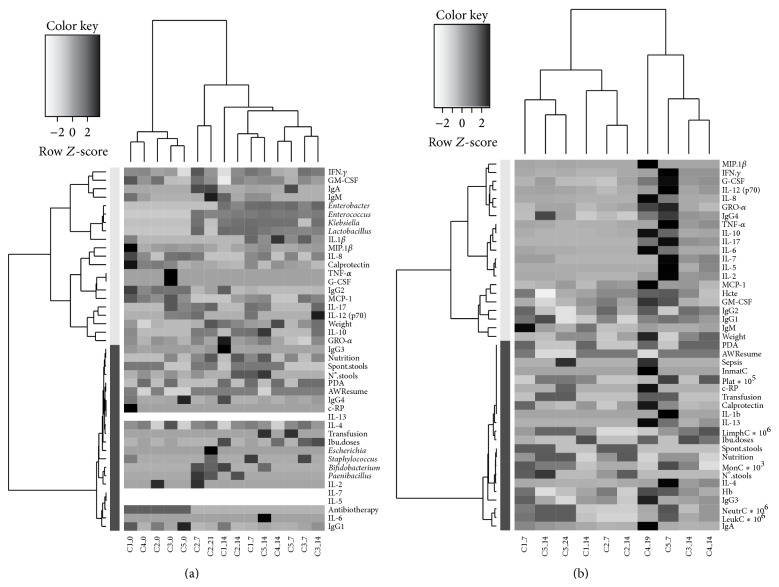
Heatmaps of fecal (a) and plasma (b) samples matrixes, considering all the quantitative variables measured and the categorized variables that were explained in the correspondent RDA, were performed. Clustering functions were applied to samples and variables after scaling the whole data set. In order to represent as much information as possible in the plot, the heatmaps were plotted using the measured data matrix scaled per variable and columns were labeled per infant and sampling time.

Those clinical categorical variables explained by the fecal and plasma RDAs were used, together with the microbiological, immunological, and clinical parameters, to create two heatmaps, one for each type of samples (Figure 4). The results from all the available fecal samples of the 5 infants were used to perform the heatmap showed in Figure 4(a). The samples' dendrogram shows two arms which clearly separate meconium and feces. The variables' dendrogram, obtained after samples clustering, shows two principal arms. The lower one is divided into two: the first of them that included clinical variables, some bacterial genera such as* Escherichia*,* Staphylococcus*,* Bifidobacterium*, and* Paenibacillus,* immunoglobulins IgG_3_ and IgG_4_, and cytokines IL-4, IL-13, and IL-2 and the second one that included antibiotherapy, IgG_1_, IL-5, IL-6, and IL-7. The upper arm is also divided and included the rest of the bacterial genera and immunological parameters together with the weight of the infants. The results obtained for all the available plasma samples from the 5 participants were used to perform the heatmap showed in Figure 4(b). The plasma samples' dendrogram shows two groups, in one of them 2 samples of the infant 2 cluster together with her twin at day 14 and samples of infant 5 clusters together with sample of infant 1 at day 7. In the second arm, siblings 3 and 4 at day 14 of probiotic supplementation initiate the clustering, which ends with sample of day 7 of infant 5 and sample of day 19 of infant 4 as previously observed in Figure 3. The dendrogram related to variables, obtained after infants clustering, showed two principal arms: one of them included clinical variables, hematological parameters, calprotectin, IL-1_*β*_, IL-4, IL-13, immunoglobulins IgA and IgG_3_, ibuprofen doses, and Hb and the second principal arm also divided including most of the cytokines, chemokines, and growth factors, the rest of the immunoglobulins, the birth weight, and the Hcte.

## 4. Discussion

In this pilot study, the bacterial composition of fecal samples obtained from five preterm infants supplemented with a probiotic mixture of two strains isolated from human milk during their earlier days of life at the NICU was assessed. In addition, a wide range of cytokines, chemokines, growth factors, and immunoglobulins were determined in all plasma, meconium, and fecal samples in order to describe their immunological profiles, their changes over time, and their potential relationship with bacterial colonization and clinical features.

The results obtained in this study suggest that the administration of* B. breve* PS12929 and* L. salivarius* PS12934 to preterm infants may increase the levels of* Lactobacillus* and* Bifidobacterium* in their feces. In fact,* L. salivarius* PS12934 could be isolated from the fecal samples of the preterm infants from day 7 of intervention and its presence remained constant throughout the study.* B. breve* PS12929 was also isolated from fecal samples after day 14 of intervention and, since then, it had increasing presence in the fecal samples. The higher frequency and concentration of* Lactobacillus* and* Bifidobacterium* in the feces analyzed should be considered a positive outcome of this study because the pattern of gut colonization in this specific infant population is usually characterized by a dominance of opportunistic pathogens and a reduced (or even absent) population of lactobacilli and bifidobacteria [7, 15, 38]. In fact, the SDI values of the fecal samples were higher than those previously described in a similar cohort that did not receive probiotics [7]. The intensive use of antibiotics at the NICU has been related to a dramatic reduction in microbial diversity and to increased presence of* Enterobacter* [39]; however, the administration of the probiotic strains in this study seemed to, somehow, compensate the antibiotic side effects.

Up to the present, there has been a complete lack of studies focused on fecal immunological parameters among preterm infants. As a consequence, there are no reference values for this population and, therefore, this study may constitute a starting point for future investigations. Although scarce, there are some studies dealing with blood immune compounds in preterm babies. Globally, they show that there are differences in the blood immune profiles depending on the infant gestational age [40–42]. It is important to note that the volume of the blood samples that are usually extracted from preterm neonates for clinical purposes is usually very low. Therefore, multiplex technologies, as the one used in this study, are required in order to be able to simultaneously analyze a high number of immune compounds [42, 43].

The results obtained in this study must be interpreted with caution due to three relevant limitations: the absence of a control group, a very small population size, and the scarcity of previous studies dealing with the immunological features of very low or extremely low weight birth infants and how they may be affected after a probiotic treatment. In this context, the levels of IL-8 found in a previous work focused on term neonates [44] were lower than those obtained in this study while those of IL-4 and IL-6 were similar; in contrast, the values of the remaining immunological parameters were higher in all the sampling times. This may illustrate the immune immaturity of these preterm infants. Similarly, levels of IL-2, IL-6, IL-8, IL-10, IL-13, IL-17, TNF-*α*, IFN-*γ*, and MCP-1 were lower in preterm infants born at 30–32 weeks than in those born after 36 weeks, indicating a lower stimulation or activation of Th1 cells and antigen-presenting cells in preterm babies as the gestational age decreases [42]. In the present work, the concentrations of the chemokines IL-8 and MCP-1 and those of the cytokines IL-4, IL-10, and IL-13, which are related to anti-inflammatory processes, were higher than those reported for preterm neonates born at 30–32 weeks and similar to those found in older infants (>36 weeks) [42]. This suggests that the administration of the probiotic strains may exert a modulatory effect on the immune system of these infants.

In addition, very low or extremely low weight birth infants usually require a strong and highly individualized medical intervention (antibiotics, oxygen, corticoids, ibuprofen, transfusions, etc.) for, at least, the first days of life due to a wide variety of life-threatening conditions. Such conditions, together with their corresponding treatments, may alter the microbial gut colonization process and, also, the infants' immune responses. Therefore, it is very difficult to obtain a homogeneous VLBW or ELBW infant population even in cohorts with a high number of infants. This is another important limitation that interventional studies, such as probiotic administration, must face when dealing with such infant subpopulations.

Despite all the limitations cited above, it is also true that a significant reduction of the inflammatory marker calprotectin in feces was observed throughout the probiotic treatment, which is in agreement with previous studies [3, 45, 46]. This is a promising outcome that must be reassessed in the future in a placebo-controlled intervention involving a large cohort.

The increase in IgA observed at day 7 may be due to the microorganisms colonizing the preterm gut, which triggers the production of this Ig by the gut-associated lymphoid tissue (GALT) [47]. IgA has the ability to penetrate the gut mucosal surface in conjunction with antigens and, as a consequence, to induce effector immune responses, playing a key role in the maintenance of intestinal microbiota and immune homeostasis [48].

The multivariate analysis applied to all the available plasma and fecal samples from the five preterm infants revealed a clear relation between the parameters assessed in this work and the clinical evolution of the infants. In the fecal-related RDA, microbial colonization acted as the principal agent opposed to the levels of certain proinflammatory immunocompounds and in agreement with the clinical variables associated with an improvement of the infants' health. Since bacterial species coordinate coefficients had positive values in the RDA1 axis, calprotectin and other proinflammatory parameters, such as IL-8, MIP-1_*β*_, MCP-1, G-CSF, or TNF-*α*, showed negative values. RDA1 axis coordinate coefficients for IgG_1_, IgG_2_, and IgG_4_ were negative while those for the secretory IgA and IgM immunoglobulins were positive. Although these findings must be taken with caution due to the inherent limitations of this work and to the high number of potential interactions and confusing factors, it should be noted that an abnormal gut microbial colonization predisposes the neonatal intestine to inflammation and to a cascade of proinflammatory and anti-inflammatory cytokines responses [49]. On the other hand, the evolution of the infants' microbiota was different than that observed in other preterm infants devoid of probiotic treatment [7] but similar to that of preterm neonates that received probiotics [23].

Finally, the dendrograms obtained for samples and variables represented in the heatmaps (Figure 4) seem to reinforce the hypothesis that probiotic strains may contribute to the development of a normal gut bacterial colonization and that this process is essential to reduce the health burden associated with prematurity [50, 51]. Although the present cohort was very small, a promising influence of the probiotic supplementation on gut colonization was observed, including an increase in bacterial diversity and in the presence of lactobacilli and bifidobacteria at relatively high levels.

Although multicenter, randomized clinical trials involving bigger cohorts and longer intervention times with these strains will be required to determine their efficacy in the prevention of sepsis or NEC, the results of this work may provide useful information for future studies dealing with probiotic gut colonization and, particularly, with the detection and quantification of fecal and blood immunocompounds in preterm infants.

## Supplementary Material

The supplementary materials include: Table S1 with additional clinical relevant data of the participants, Table S2 that includes the comparison between the frequencies and concentrations of all the immune compounds measured in all plasma and fecal samples and finally, Figure S1 that shown the heatmap of all the bacterial species found in the fecal samples of this study.

## Figures and Tables

**Table 1:** Epidemiological and clinical relevant data from the mother-infant pairs of this study.

Mothers	1		2		3
Age (years)	30		18		28
Fever	No		Yes		No
Leukocytosis (>15,000 leukocytes/*µ*L)	No		Yes		Yes
C-reactive protein (mg/L)	26		7.6		40
Antenatal antibiotics treatment	Yes		Yes		Yes
Antenatal corticosteroids treatment	Complete		Uncomplete		Complete
Chorioamnionitis	No		Yes		Yes
Type of delivery	C-section		C-section		C-section
Multiple delivery	Yes		Yes		No

Infants	1	2	3	4	5

Rupture of fetal membranes (h)	672	0	0	0	432
Twin position	1	2	2	1	1
Sex	F	F	F	F	F
Gestational age (wk)	28 + 5	28 + 5	28 + 6	28 + 6	27 + 2
Birth weight (g) (*Z*-score)	1070 (−0.71)	980 (1.01)	1082 (−0.66)	1200 (−0.26)	770 (−1.02)
Birth height (cm) (*Z*-score)	36 (−1.3)	36 (−1.3)	36 (−1.3)	36 (−1.3)	32 (−1.8)
Birth head circumference (cm) (*Z*-score)	26 (−0.8)	26 (−0.8)	25.5 (−1.1)	26 (−0.8)	24 (−0.8)
Apgar score at 1 min	8	9	8	5	7
Apgar score at 5 min	9	9	9	7	8
Revival	Ventilation	No	Ventilation	Ventilation	Ventilation
PDA	Yes	No	Yes	Yes	No
Meconium spontaneous expulsion	Yes	Yes	Yes	Yes	Yes
Meconium expulsion (h)	24	9	48	36	14
Probiotic starting age (d)	2	2	2	2	4
Probiotic treatment length (d)	18	18	31	19	25
NICU stay (d)	18	8	14	64	49
Age at discharge (d)	51	51	60	64	101
Corrected gestational age at discharge (wk)	36	36	37	38	42
Death	No	No	No	Yes	No

PDA: patent ductus arteriosus; NICU: neonatal intensive care unit.

Antenatal corticosteroid treatment was uncompleted or complete when mother received one or two doses of betamethasone, respectively, within one week and 24 h before delivery.

Apgar test ranged from 1 to 10: less than 5 means risk; up to 7 means normal.

Twin position means the position at birth, 1 being the infant who was nearest to the cervix.

**Table 2:** Frequency and concentration of immune compounds in fecal samples (*N* = 14) along time.

	Day 0 (*N* = 5)		Day 7 (*N* = 4)		Day 14 (*N* = 5)		*P* value^*^
	*n* (%)	Median (IQR)	*n* (%)	Median (IQR)	*n* (%)	Median (IQR)	
		(mg/kg)		(mg/kg)		(mg/kg)	
Immunoglobulins							
IgG_1_	5 (100)	3.95 (1.23–6.36)	4 (100)	0.45 (0.23–0.80)	5 (100)	1.26 (0.47–2.43)	0.819
IgG_2_	5 (100)	23.82 (23.19–24.17)	4 (100)	2.98 (2.46–3.97)	5 (100)	2.66 (2.60–3.62)	0.074
IgG_3_	4 (80)	0.02 (0.01–0.02)	1 (25)	0.01 (0.01–0.01)	2 (40)	0.22 (0.11–0.32)	0.424
IgG_4_	5 (100)	0.03 (0.02–0.14)	4 (100)	0.02 (0.01–0.03)	5 (100)	0.03 (0.00–0.06)	0.449
IgM	4 (80)	2.72 (0.19–8.73)	3 (75)	1.10 (0.87–6.00)	5 (100)	2.79 (0.44–10.02)	0.819
IgA	5 (100)	3.57 (0.88–21.73)	4 (100)	201.23 (35.09–356.74)	5 (100)	7.49 (2.96–7.78)	0.074

		(ng/kg)		(ng/kg)		(ng/kg)	

Proinflammatory							
Calprotectin^†^	5 (100)	309.50 (282.00–343.90)	4 (100)	144.80 (132.30–180.40)	5 (100)	38.42 (34.16–63.96)	0.041
IL-1_β_^‡^	1 (20)	31.47	3 (75)	41.34 (8.00–74.68)	3 (60)	39.20 (−36.24–114.64)	0.937
IL-2	1 (20)	8.47	1 (25)	8.18	0 (0)	—	0.368
IL-6	0 (0)	—	0 (0)	—	1 (20)	27.44	0.368
IL-12 (p70)	2 (40)	29.07 (28.82–29.32)	2 (50)	37.13 (36.38–37.89)	1 (20)	82.98	0.926
IL-17	2 (40)	72.94 (62.76–83.11)	2 (50)	66.08 (64.46–67.71)	2 (40)	69.31 (65.15–73.48)	1.000
IFN-*γ*	4 (80)	214.90 (190.40–238.30)	4 (100)	299.80 (255.40–320.80)	4 (80)	248.10 (215.80–265.50)	0.449
TNF-*α*	1 (20)	20.87	0 (0)	—	0 (0)	—	0.368

		(ng/kg)		(ng/kg)		(ng/kg)	

Anti-inflammatory							
IL-4	3 (60)	2.74 (2.43–3.48)	4 (100)	2.63 (2.49–2.85)	3 (60)	2.12 (2.06–2.26)	0.268
IL-5	0 (0)	—	0 (0)	—	0 (0)	—	—
IL-10	1 (20)	25.62	2 (50)	37.21 (35.85–38.57)	3 (60)	39.20 (38.66–53.87)	0.319
IL-13	0 (0)	—	0 (0)	—	0 (0)	—	—

		(ng/kg)		(ng/kg)		(ng/kg)	

Chemokines							
IL-8	4 (80)	20.94 (19.00–23.82)	3 (75)	16.16 (15.56–17.20)	2 (40)	17.05 (16.45–17.64)	0.128
GRO-*α* ^‡^	5 (100)	206.30 (117.04–295.57)	3 (75)	222.10 (−77.61–521.80)	4 (80)	263.50 (261.05–265.88)	0.763
MCP-1	5 (100)	20.08 (15.02–28.89)	2 (50)	18.37 (16.82–19.93)	3 (60)	16.98 (14.21–17.34)	0.076
MIP-1_*β*_	5 (100)	53.79 (52.03–68.66)	4 (100)	58.16 (46.46–66.42)	4 (80)	49.60 (35.16–69.89)	0.449

		(ng/kg)		(ng/kg)		(ng/kg)	

Haematopoietic stimuli							
IL-7	0 (0)	—	0 (0)	—	0 (0)	—	—
G-CSF	1 (20)	28.99	0 (0)	—	0 (0)	—	0.368
GM-CSF	5 (100)	1729.00 (1086.00–2312.00)	4 (100)	1830.00 (1648.00–2010.00)	4 (80)	1879.00 (1783.00–1920.00)	0.819

Levels of immune compounds were expressed as median and interquartile range (IQR) when data were not normally distributed and as mean and 95% confidence interval (95% CI) when they were. ^*^Friedman test was used to determine the differences between fecal samples along time when data were not normally distributed and one-way ANOVA when they were. ^†^Concentration was expressed as ng/Kg of feces for all the proinflammatory parameters with the exception of calprotectin whose units were mg/Kg. ^‡^Normally distributed.

**Table 3:** Frequency and concentration of immune compounds in plasma samples (*N* = 8) along time.

	Day 7 (*N* = 3)		Day 14 (*N* = 5)		*P* value^*^
	*n* (%)	Median (IQR)	*n* (%)	Median (IQR)	
		(mg/L)		(mg/L)	
Immunoglobulins					
IgG_1_	3 (100)	2159.80 (2075.95–2174.30)	5 (100)	1727.30 (1205.50–2029.60)	0.297
IgG_2_	3 (100)	1135.20 (796.03–1147.50)	5 (100)	741.24 (683.84–930.95)	0.456
IgG_3_	3 (100)	52.54 (46.75–64.53)	5 (100)	43.91 (41.35–48.14)	0.297
IgG_4_	3 (100)	23.25 (22.02–67.30)	5 (100)	44.66 (10.59–49.08)	0.655
IgM	3 (100)	263.75 (176.71–934.18)	5 (100)	335.18 (261.41–366.78)	0.882
IgA	3 (100)	27.03 (18.20–40.41)	5 (100)	4.44 (4.00–14.31)	0.101

		(ng/L)		(ng/L)	

Proinflammatory					
Calprotectin^†^	3 (100)	0.86 (0.47–1.11)	5 (100)	0.37 (0.37–0.63)	0.456
IL-1_β_^‡^	1 (33)	15.81	0 (0)	—	—
IL-2	2 (67)	35.41 (19.34–51.48)	3 (60)	9.70 (6.54–11.23)	1.000
IL-6	3 (100)	24.14 (15.99–65.65)	5 (100)	17.06 (10.10–19.24)	0.297
IL-12 (p70)	3 (100)	27.55 (19.89–91.22)	5 (100)	28.35 (22.71–29.16)	0.882
IL-17	1 (33)	167.20	2 (40)	35.66 (34.65–36.67)	0.221
IFN-*γ*	2 (67)	670.07 (371.30–968.83)	4 (80)	150.06 (67.73–225.91)	0.643
TNF-*α*	3 (100)	15.06 (11.35–66.01)	5 (100)	13.14 (11.83–20.40)	0.764

		(ng/L)		(ng/L)	

Anti-inflammatory					
IL-4	3 (100)	1.95 (1.57–7.96)	5 (100)	1.99 (1.69–2.90)	0.882
IL-5	1 (33)	39.43	1 (20)	9.65	0.317
IL-10	3 (100)	11.80 (11.10–69.56)	3 (60)	20.11 (16.02–22.68)	0.513
IL-13	1 (33)	11.27	1 (20)	5.06	0.317

		(ng/L)		(ng/L)	

Chemokines					
IL-8	3 (100)	31.69 (24.45–85.28)	5 (100)	29.76 (22.37–30.79)	0.655
GRO-*α* ^‡^	2 (67)	204.44 (−1859.94–2268.82)	3 (60)	55.42 (45.11–65.73)	0.306
MCP-1	3 (100)	193.91 (123.14–204.59)	5 (100)	88.62 (60.77–192.54)	0.456
MIP-1_*β*_	3 (100)	234.90 (210.30–292.20)	5 (100)	174.60 (150.00–250.80)	0.297

		(ng/L)		(ng/L)	

Haematopoietic stimuli					
IL-7	2 (67)	28.14 (17.72–38.57)	3 (60)	10.48 (8.84–12.50)	0.564
G-CSF	3 (100)	30.89 (23.21–96.81)	5 (100)	47.27 (41.84–51.46)	0.655
GM-CSF	3 (100)	248.80 (191.00–299.30)	4 (80)	132.35 (114.97–151.83)	0.157

Levels of immune compounds were expressed as median and interquartile range (IQR) when data were not normally distributed and as mean and 95% confidence interval (95% CI) when they were. ^*^Kruskal-Wallis test was used to determine the differences between blood samples along time when data were not normally distributed and one-way ANOVA test when they were. ^†^Concentration was expressed as ng/L of plasma for all the proinflammatory parameters with the exception of calprotectin whose units were mg/L. ^‡^Normally distributed.

## References

[1] Magne F., Abély M., Boyer F., Morville P., Pochart P., Suau A. (2006). Low species diversity and high interindividual variability in faeces of preterm infants as revealed by sequences of 16S rRNA genes and PCR-temporal temperature gradient gel electrophoresis profiles. *FEMS Microbiology Ecology*.

[2] Wang Y., Hoenig J. D., Malin K. J. (2009). 16S rRNA gene-based analysis of fecal microbiota from preterm infants with and without necrotizing enterocolitis. *ISME Journal*.

[3] Rougé C., Goldenberg O., Ferraris L. (2010). Investigation of the intestinal microbiota in preterm infants using different methods. *Anaerobe*.

[4] LaTuga M. S., Ellis J. C., Cotton C. M. (2011). Beyond bacteria: a study of the enteric microbial consortium in extremely low birth weight infants. *PLoS ONE*.

[5] Arboleya S., Binetti A., Salazar N. (2012). Establishment and development of intestinal microbiota in preterm neonates. *FEMS Microbiology Ecology*.

[6] Hallab J. C., Leach S. T., Zhang L. (2013). Molecular characterization of bacterial colonization in the preterm and term infant's intestine. *Indian Journal of Pediatrics*.

[7] Moles L., Gómez M., Heilig H. (2013). Bacterial diversity in meconium of preterm neonates and evolution of their fecal microbiota during the first month of life. *PLoS ONE*.

[8] Martín R., Nauta A. J., Ben Amor K., Knippels L. M., Knol J., Garssen J. (2010). Early life: gut microbiota and immune development in infancy. *Beneficial microbes*.

[9] Kaplan J. L., Shi H. N., Walker W. A. (2011). The role of microbes in developmental immunologic programming. *Pediatric Research*.

[10] van Baarlen P., Wells J. M., Kleerebezem M. (2013). Regulation of intestinal homeostasis and immunity with probiotic lactobacilli. *Trends in Immunology*.

[11] Sharma A. A., Jen R., Butler A., Lavoie P. M. (2012). The developing human preterm neonatal immune system: a case for more research in this area. *Clinical Immunology*.

[12] Claud E. C., Walker W. A. (2001). Hypothesis: inappropriate colonization of the premature intestine can cause neonatal necrotizing enterocolitis. *The FASEB Journal*.

[13] de la Cochetière M.-F., Piloquet H., des Robert C., Darmaun D., Galmiche J.-P., Rozé J.-C. (2004). Early intestinal bacterial colonization and necrotizing enterocolitis in premature infants: the putative role of *Clostridium*. *Pediatric Research*.

[14] Butel M.-J., Suau A., Campeotto F. (2007). Conditions of bifidobacterial colonization in preterm infants: a prospective analysis. *Journal of Pediatric Gastroenterology and Nutrition*.

[15] Madan J. C., Salari R. C., Saxena D. (2012). Gut microbial colonisation in premature neonates predicts neonatal sepsis. *Archives of Disease in Childhood: Fetal and Neonatal Edition*.

[16] Janvier A., Malo J., Barrington K. J. (2014). Cohort study of probiotics in a North American neonatal intensive care unit. *Journal of Pediatrics*.

[17] Embleton N. D., Skeath T. (2014). Probiotics for preterm infants on the NICU. *Paediatrics and Child Health*.

[18] Wang Q., Dong J., Zhu Y. (2012). Probiotic supplement reduces risk of necrotizing enterocolitis and mortality in preterm very low-birth-weight infants: an updated meta-analysis of 20 randomized, controlled trials. *Journal of Pediatric Surgery*.

[19] Deshpande G., Rao S., Patole S., Bulsara M. (2010). Updated meta-analysis of probiotics for preventing necrotizing enterocolitis in preterm neonates. *Pediatrics*.

[20] Tarnow-Mordi W. O., Wilkinson D., Trivedi A., Brok J. (2010). Probiotics reduce all-cause mortality and necrotizing enterocolitis: it is time to change practice. *Pediatrics*.

[21] Lin H.-C., Hsu C.-H., Chen H.-L. (2008). Oral probiotics prevent necrotizing enterocolitis in very low birth weight preterm infants: a multicenter, randomized, controlled trial. *Pediatrics*.

[22] Romeo M. G., Romeo D. M., Trovato L. (2011). Role of probiotics in the prevention of the enteric colonization by *Candida* in preterm newborns: Incidence of late-onset sepsis and neurological outcome. *Journal of Perinatology*.

[23] Mohan R., Koebnick C., Schildt J. (2006). Effects of *Bifidobacterium lactis* Bb12 supplementation on intestinal microbiota of preterm infants: a double-blind, placebo-controlled, randomized study. *Journal of Clinical Microbiology*.

[24] Ohashi Y., Ushida K. (2009). Health-beneficial effects of probiotics: its mode of action. *Animal Science Journal*.

[25] Millar M., Wilks M., Fleming P., Costeloe K. (2012). Should the use of probiotics in the preterm be routine?. *Archives of Disease in Childhood: Fetal and Neonatal Edition*.

[26] Cristofalo E. A., Schanler R. J., Blanco C. L. (2013). Randomized trial of exclusive human milk versus preterm formula diets in extremely premature infants. *The Journal of Pediatrics*.

[27] Ahrabi A. F., Schanler R. J. (2013). Human milk is the only milk for premies in the NICU!. *Early Human Development*.

[28] Tudehope D. I. (2013). Human milk and the nutritional needs of preterm infants. *Journal of Pediatrics*.

[29] Levy I., Comarsca J., Davidovits M., Klinger G., Sirota L., Linder N. (2009). Urinary tract infection in preterm infants: the protective role of breastfeeding. *Pediatric Nephrology*.

[30] Schanler R. J., Lau C., Hurst N. M., Smith E. O. (2005). Randomized trial of donor human milk versus preterm formula as substitutes for mothers' own milk in the feeding of extremely premature infants. *Pediatrics*.

[31] Martín R., Olivares M., Marín M. L., Fernández L., Xaus J., Rodríguez J. M. (2005). Probiotic potential of 3 lactobacilli strains isolated from breast milk. *Journal of Human Lactation*.

[32] Martín R., Jiménez E., Heilig H. (2009). Isolation of bifidobacteria from breast milk and assessment of the bifidobacterial population by PCR-denaturing gradient gel electrophoresis and quantitative real-time PCR. *Applied and Environmental Microbiology*.

[33] Fernández L., Langa S., Martín V. (2013). The human milk microbiota: origin and potential roles in health and disease. *Pharmacological Research*.

[34] Gueimonde M., Laitinen K., Salminen S., Isolauri E. (2007). Breast milk: a source of bifidobacteria for infant gut development and maturation?. *Neonatology*.

[35] Solís G., de los Reyes-Gavilan C. G., Fernández N., Margolles A., Gueimonde M. (2010). Establishment and development of lactic acid bacteria and bifidobacteria microbiota in breast-milk and the infant gut. *Anaerobe*.

[36] Arboleya S., Ruas-Madiedo P., Margolles A. (2011). Characterization and *in vitro* properties of potentially probiotic *Bifidobacterium* strains isolated from breast-milk. *International Journal of Food Microbiology*.

[37] Martín V., Maldonado-Barragán A., Moles L. (2012). Sharing of bacterial strains between breast milk and infant feces. *Journal of Human Lactation*.

[38] Mshvildadze M., Neu J., Shuster J., Theriaque D., Li N., Mai V. (2010). Intestinal microbial ecology in premature infants assessed with non-culture-based techniques. *Journal of Pediatrics*.

[39] Greenwood C., Morrow A. L., Lagomarcino A. J. (2014). Early empiric antibiotic use in preterm infants is associated with lower bacterial diversity and higher relative abundance of *Enterobacter*. *The Journal of Pediatrics*.

[40] Blanco-Quirós A., Arranz E., Solis G., Villar A., Ramos A., Coto D. (2000). Cord blood interleukin-10 levels are increased in preterm newborns. *European Journal of Pediatrics*.

[41] Matoba N., Yu N., Mestan K. (2009). Differential patterns of 27 cord blood immune biomarkers across gestational age. *Pediatrics*.

[42] Lusyati S., Hulzebos C. V., Zandvoort J., Sukandar H., Sauer P. J. J. (2013). Cytokines patterns in newborn infants with late onset sepsis. *Journal of Neonatal-Perinatal Medicine*.

[43] Takahashi N., Uehara R., Kobayashi M. (2010). Cytokine profiles of seventeen cytokines, growth factors and chemokines in cord blood and its relation to perinatal clinical findings. *Cytokine*.

[44] Hodge G., Hodge S., Haslam R. (2004). Rapid simultaneous measurement of multiple cytokines using 100 *μ*l sample volumes—Association with neonatal sepsis. *Clinical and Experimental Immunology*.

[45] Josefsson S., Bunn S. K., Domellöf M. (2007). Fecal calprotectin in very low birth weight infants. *Journal of Pediatric Gastroenterology and Nutrition*.

[46] Aydemir G., Cekmez F., Tanju I. A. (2012). Increased fecal calprotectin in preterm infants with necrotizing enterocolitis. *Clinical Laboratory*.

[47] Suzuki K., Ha S.-A., Tsuji M., Fagarasan S. (2007). Intestinal IgA synthesis: a primitive form of adaptive immunity that regulates microbial communities in the gut. *Seminars in Immunology*.

[48] Rogosch T., Kerzel S., Hoß K. (2012). IgA response in preterm neonates shows little evidence of antigen-driven selection. *The Journal of Immunology*.

[49] Mai V., Torrazza R. M., Ukhanova M. (2013). Distortions in development of intestinal microbiota associated with late onset sepsis in preterm infants. *PLoS ONE*.

[50] Mai V., Young C. M., Ukhanova M. (2011). Fecal microbiota in premature infants prior to necrotizing enterocolitis. *PLoS ONE*.

[51] Siggers R. H., Siggers J., Thymann T., Boye M., Sangild P. T. (2011). Nutritional modulation of the gut microbiota and immune system in preterm neonates susceptible to necrotizing enterocolitis. *Journal of Nutritional Biochemistry*.

