# Supplementary material for: Administration of Bifidobacterium breve PS12929 and Lactobacillus salivarius PS12934, Two Strains Isolated from Human Milk, to Very Low and Extremely Low Birth Weight Preterm Infants: A Pilot Study
Source: J Immunol Res. 2015 Feb 22;2015:538171. doi: 10.1155/2015/538171 (PMC4352454; doi:10.1155/2015/538171)
Supplement: Supplementary file 1 — The supplementary materials include: Table S1 with additional clinical relevant data of the participants, Table S2 that includes the comparison between the frequencies and concentrations of all the immune compounds measured in all plasma and fecal samples and finally, Figure S1 that shown the heatmap of all the bacterial species found in the fecal samples of this study. [file 538171.f1.zip › SupMaterialDemographic_and_clinical_data.docx]

***Demographic and clinical characteristics of the participants***

Mothers

The mother of twins 1 and 2 presented a high level of C-reactive protein (C-RP) and premature rupture of the membranes (PROM) in infant 1, so was treated with intravenous antibiotics (ampicillin (1 g/6 h), gentamicin (240 mg/24 h) and azithromycin (500 mg/12 h)) for at least 672 h before delivery.

The mother of siblings 3 and 4 was the younger one (18 years old) and suffered fever during pregnancy and chorioamnionitis that caused the premature delivery. Maternal antibiotherapy consisted in a combination of ampicillin (1 g/6 h), gentamicin (240 mg/24 h) and azithromycin (500 mg/12 h) around 24 h before delivery.

Mother of infant 5 suffered gestational diabetes and depression. She also experienced chorioamnionitis with high C-RP levels and PROM that caused the premature delivery. Maternal antibiotherapy consisted in a combination of ampicillin (1 g/6 h), gentamicin (240 mg/24 h) and azithromycin (500 mg/12 h) for 432 h before delivery.

All the mothers were treated with corticosteroids to improve the fetus maturation, although only two of them completed the treatment receiving two doses of betametasone.

Infants

Infants 1 and 2 were treated with ampicillin (50 mg/kg/12 h), gentamicin (5 mg/kg/48 h) and also fluconazol (3 mg/kg/72 h) during the first days of life (antifungal treatment was longer in infant 1). The clinical evolution was good although more interventions such as mechanical ventilation and ibuprofen treatment for PDA were required in infant 1 (Table 1). Both infants started with parenteral nutrition and was changed to enteral nutrition as soon as possible with mixed feeding including own mother’s milk during NICU stay. Microbiological and immunological parameters in infant 2 were in accordance with the mean values obtain in the study. However infant 1 presented higher amount of staphylococci (from meconium) and *Proteobacteria* at first week of intervention. In both infants, the highest concentrations of *B. breve* and *L. salivarius* were detected at all the collection points, which could be related with the use of human milk. Considering immunological parameters infant 1 had high values of Calprotectin at birth and considerably low values of most of proinflammatory components at day 14^th^ both in plasma and feces.

Infants 3 and 4 required mechanical ventilation, ibuprofen for PDA treatment (longer in infant 4) and empiric antibiotic treatment of a combination of ampicillin, gentamicin and clarithromycin (10 mg/kg/12 h). Parenteral nutrition and trophic enteral feeding started at birth; but owns mother’s milk was not available. Enteral feeding of infant 3 was interrupted at day 7, but rapidly restituted and at day 28 achieved full enteral feeding. Infant 4 suffered a gastric bleeding at day 7 despite a good weight acquisition and continued with trophic enteral feeding until day 14 when it was completely interrupted due to apnea pauses and a big ductus that entailed ibuprofen treatment. At day 17^th^ of life (15^th^ of study) infection symptoms were detected, thus a combination of amikacin (14 mg/kg/24 h) and vancomycin (10 mg/kg/12 h) was prescribed. Positive blood culture of *Enterobacter kobei* and high C-RP levels were observed at day 19. At this moment infant 4 was excluded from the study, although an epicutaneous jugular catheter was identified as the focus of the infection developing such a serious septicemia that caused the infant’s death at the age of 2 months by ascites (fluid accumulation in the peritoneal cavity) and liver failure. However clinical, microbiological and immunological progress of both infants was very similar during the first weeks of life (with the exception of 7^th^ day of immunological compounds). Infant 3 present immune components in accordance with the study with the exception of elevated IL-6 in meconium sample and infant 4 presented high calprotectin at day 14^th^. Also most of the studied parameters such as calprotectin, the majority of immunoglobulins, pro-inflammatory compounds as IL-1_β_, IL-6, IL-12 and IL-17, anti-inflammatory compounds IL-10 and IL-13, and all the chemokines, were higher than the obtained mean value in plasma sample of day 19^th^ in infant 4.

The infant 5 required ventilation and also empiric antibiotherapy of ampicillin, gentamicin and clarithromycin in addition to antifungal therapy with fluconazol. Trophic enteral feeding started at birth and progressively increased raising full enteral nutrition at day 14 of intervention, although her mother’s milk was not available. Clinical evolution was good although at day 12 of treatment a hematite’s transfusion was required. Symptoms of infection were detected at day 20 of probiotic treatment and a combination of amikacin and vancomycin was prescribed. High levels of C-RP and abdominal distention with pain were present at day 28, antibiotic treatment was changed to amikacin and cefepime (50 mg/kg/12 h) and clinical evolution was favorable. Positive cultures of *Enterococcus faecalis*, *Enterobacter cloacae* and *Klebsiella pneumoniae* were obtained. The microorganisms causing the septicemia in infant 5 were isolated in high concentrations from fecal samples; at day 7^th^ *E. faecalis* and *E. cloacae,* being the last one decreased considerably at day 14^th^ but with the appearance of *K. pneumoniae* in high concentrations at this sampling time. After sepsis treatment, those microorganisms considerably decreased (or even disappeared) and beneficial bacteria (*B. breve*) increased. Plasma samples of infant 5 at days 24^th^ showed low values of immunoglobulins with the exception of IgA (elevated also in fecal samples) and IgG_4_ (plasma). Pro-inflammatory, anti-inflammatory and haematopoietic stimule compounds and chemiokines IL-8 and GROα had elevated values at day 7^th^ in plasma samples and some of them (IFNγ and TNFα) present low values at day 24.
