# Supplementary material for: Administration of Bifidobacterium breve PS12929 and Lactobacillus salivarius PS12934, Two Strains Isolated from Human Milk, to Very Low and Extremely Low Birth Weight Preterm Infants: A Pilot Study
Source: J Immunol Res. 2015 Feb 22;2015:538171. doi: 10.1155/2015/538171 (PMC4352454; doi:10.1155/2015/538171)
Supplement: Supplementary file 1 — The supplementary materials include: Table S1 with additional clinical relevant data of the participants, Table S2 that includes the comparison between the frequencies and concentrations of all the immune compounds measured in all plasma and fecal samples and finally, Figure S1 that shown the heatmap of all the bacterial species found in the fecal samples of this study. [file 538171.f1.zip › Table S1.docx]

**Table S1:** Antibiotherapy, Sepsis development and treatment, nutrition features and PDA treatment of each Infant.

|  | **Infant 1** | **Infant 2** | **Infant 3** | **Infant 4** | **Infant 5** |
| --- | --- | --- | --- | --- | --- |
| **Antibiotherapy** |  |  |  |  |  |
| **Ampicillin (50 mg/Kg/12h)** | Yes | Yes | Yes | Yes | Yes |
| **Clarithromycin (10 mg/Kg/12h)** | No | No | No | Yes | Yes |
| **Fluconazol (3 mg/Kg/72h)** | Yes | Yes | No | No | Yes |
| **Gentamicin (5 mg/Kg/48h)** | Yes | Yes | Yes | Yes | Yes |
| **Sepsis** | No | No | No | Yes | Yes |
| **Sepsis treatment** |  |  |  |  |  |
| **Amikacin (14 mg/kg/24 h)** | No | No | No | Yes | Yes |
| **Cefepime (50 mg/kg/12 h)** | No | No | No | No | Yes |
| **Vancomycin (10 mg/kg/12 h)** | No | No | No | Yes | Yes |
| **Nutrition Type** |  |  |  |  |  |
| **Week 1** | Parenteral | Parenteral | Enteral (F) | Enteral (F) | Parenteral + Trophic |
| **Week 2** | Parenteral + Trophic | Enteral (F + MM) | Parenteral | Parenteral + Trophic | Parenteral + Trophic |
| **Week 3** | Parenteral + Trophic | Enteral (F + MM) | Parenteral | Parenteral | Enteral (F) |
| **Week 4** | Enteral (F + MM) | Enteral (F + MM) | Parenteral | Parenteral | Enteral (F) |
| **PDA treatment** | Yes | No | Yes | Yes | No |
| **Dose 1 (Ibuprofen 10mg/kg)** | Yes |  | Yes | Yes |  |
| **Dose 2 (Ibuprofen 5mg/kg)** | Yes |  | Yes | Yes |  |
| **Dose 3 (Ibuprofen 5mg/kg)** | Yes |  | Yes | Yes |  |
| **Dose 4 (Ibuprofen 5mg/kg)** | Yes |  | Yes | Yes |  |
| **Dose 5 (Ibuprofen 5mg/kg)** | Yes |  | Yes | Yes |  |
| **Dose 6 (Ibuprofen 5mg/kg)** | Yes |  | Yes | No |  |

Tropic means around 30mL/day of formula feeding

F: Formula feeding

MM: Mother milk feeding
