# Supplementary material for: Administration of Bifidobacterium breve PS12929 and Lactobacillus salivarius PS12934, Two Strains Isolated from Human Milk, to Very Low and Extremely Low Birth Weight Preterm Infants: A Pilot Study
Source: J Immunol Res. 2015 Feb 22;2015:538171. doi: 10.1155/2015/538171 (PMC4352454; doi:10.1155/2015/538171)
Supplement: Supplementary file 1 — The supplementary materials include: Table S1 with additional clinical relevant data of the participants, Table S2 that includes the comparison between the frequencies and concentrations of all the immune compounds measured in all plasma and fecal samples and finally, Figure S1 that shown the heatmap of all the bacterial species found in the fecal samples of this study. [file 538171.f1.zip › Table S2.docx]

**Table S2:** Frequency and concentration of immune compounds in all plasma (N=8) and fecal (N=15) samples analyzed in this study

|  |  | **Plasma (N=8)** | | **Feces (N=15)** | | **p-value*** |
| --- | --- | --- | --- | --- | --- | --- |
|  |  | **n (%)** | **Median (IQR)** | **n (%)** | **Median (IQR)** |  |
| ***Immunoglobulins*** | |  | **(mg/L)** |  | **(mg/Kg)** |  |
| **IgG_1_** |  | 8 (100) | 2010.85 (1596.85 - 2167.05) | 15 (100) | 1.23 (0.45 - 3.19) | 0.000 |
| **IgG_2_** |  | 8 (100) | 836.09 (644.00 - 1108.65) | 15 (100) | 5.72 (2.63 - 23.50) | 0.000 |
| **IgG_3_** |  | 8 (100) | 46.03 (41.25 - 51.47) | 8 (53) | 0.02 (0.01 - 0.03) | 0.000 |
| **IgG_4_** |  | 8 (100) | 33.96 (18.23 - 62.56) | 15 (100) | 0.03 (0.01 - 0.05) | 0.000 |
| **IgM** |  | 8 (100) | 299.46 (230.66 - 455.02) | 13 (87) | 2.79 (0.44 - 10.91) | 0.000 |
| **IgA** |  | 8 (100) | 11.85 (4.33 - 28.11) | 15 (100) | 7.78 (1.92 - 39.48) | 0.897 |
| ***Pro-inflammatory*** | |  | **(ng/L)** |  | **(ng/Kg)** |  |
| **Calprotectin** |  | 8 (100) | 0.50 (0.35 - 0.74) | 18/19 | 144.85 (74.39 - 302.62) | 0.000 |
| **IL-1_β_**^‡^ |  | 1 (13) | 15.81 | 7 (47) | 39.01 (20.99 - 57.03) | 0.308 |
| **IL-2** |  | 5 (63) | 9.70 (3.39 - 12.76) | 2 (13) | 8.32 (8.25 - 8.40) | 0.698 |
| **IL-6** |  | 8 (100) | 18.15 (9.54 - 20.74) | 1 (7) | 27.44 | 0.245 |
| **IL-12(p70)** |  | 8 (100) | 27.95 (22.31 - 31.18) | 5 (33) | 35.62 (29.57 - 38.65) | 0.107 |
| **IL-17** |  | 3 (37) | 37.68 (35.66 - 102.45) | 7 (47) | 69.33 (61.91 - 78.11) | 0.425 |
| **IFNγ** |  | 6 (75) | 150.06 (73.44 - 229.82) | 13 (87) | 235.00 (194.90 - 278.40) | 0.160 |
| **TNFα** |  | 8 (100) | 14.10 (10.78 - 22.70) | 1 (7) | 20.87 | 0.437 |
| ***Anti-inflammatory*** | |  | **(ng/L)** |  | **(ng/Kg)** |  |
| **IL-4** |  | 8 (100) | 1.97 (1.66 - 3.22) | 11 (73) | 2.52 (2.26 - 2.88) | 0.215 |
| **IL-5** |  | 2 (25) | 24.54 (17.09 - 31.98) | 0 (0) | - | - |
| **IL-10** |  | 6 (75) | 16.02 (11.84 - 23.97) | 7 (47) | 38.11 (33.23 - 39.56) | 0.045 |
| **IL-13** |  | 2 (25) | 8.16 (6.61 - 9.72) | 0 (0) | - | - |
| ***Chemokines*** | |  | **(ng/L)** |  | **(ng/Kg)** |  |
| **IL-8** |  | 8 (100) | 30.27 (21.68 - 33.02) | 9 (60) | 18.24 (15.86 - 20.34) | 0.012 |
| **GROα**^‡^ |  | 5 (63) | 115.03 (-59.99 - 290.05) | 13 (87) | 226.39 (178.18 - 274.60) | 0.048 |
| **MCP-1** |  | 8 (100) | 140.58 (58.67 - 197.32) | 10 (67) | 17.34 (15.08 - 23.13) | 0.000 |
| **MIP-1_β_** |  | 8 (100) | 210.30 (168.50 - 254.60) | 14 (93) | 52.91 (36.48 - 67.87) | 0.001 |
| ***Haematopoietic stimuli*** | |  | **(ng/L)** |  | **(ng/Kg)** |  |
| **IL-7** |  | 5 (63) | 10.48 (7.30 - 14.53) | 0 (0) | - | - |
| **G-CSF** |  | 8 (100) | 44.55 (28.66 - 53.18) | 1 (7) | 28.99 | 0.439 |
| **GM-CSF** |  | 7 (87) | 141.27 (128.26 - 216.17) | 14 (93) | 1848.00 (1591.00 - 1970.00) | 0.000 |

Levels of immune compounds were expressed as median and interquartile range (IQR) when data were not normal distributed and as mean and 95% confidence interval (95% CI) when they were.

^*^ Kruskal –Wallis was used to determine the differences between blood samples and fecal samples when data were not normal distributed and One-way ANOVA test when they were.

**^†^** Concentration was expressed as ng/L of plasma or ng/Kg of feces for all the pro-inflammatory parameters with the exception of calprotectin which units were mg/L for plasma and mg/Kg for feces.

^‡^Normal distributed.
